# *Lactobacillus paracasei* modulates the immune system of *Galleria mellonella* and protects against *Candida albicans* infection

**DOI:** 10.1371/journal.pone.0173332

**Published:** 2017-03-07

**Authors:** Rodnei Dennis Rossoni, Beth Burgwyn Fuchs, Patrícia Pimentel de Barros, Marisol dos Santos Velloso, Antonio Olavo Cardoso Jorge, Juliana Campos Junqueira, Eleftherios Mylonakis

**Affiliations:** 1 Department of Biosciences and Oral Diagnosis, Univ Estadual Paulista/UNESP, São José dos Campos, São Paulo, Brazil; 2 Division of Infectious Diseases, Rhode Island Hospital, Alpert Medical School of Brown University, Providence, Rhode Island, United States of America; Agricultural University of Athens, GREECE

## Abstract

Probiotics have been described as a potential strategy to control opportunistic infections due to their ability to stimulate the immune system. Using the non-vertebrate model host *Galleria mellonella*, we evaluated whether clinical isolates of *Lactobacillus* spp. are able to provide protection against *Candida albicans* infection. Among different strains of *Lactobacillus paracasei*, *Lactobacillus rhamnosus* and *Lactobacillus fermentum*, we verified that *L*. *paracasei* 28.4 strain had the greatest ability to prolong the survival of larvae infected with a lethal dose of *C*. *albicans*. We found that the injection of 10^7^ cells/larvae of *L*. *paracasei* into *G*. *mellonella* larvae infected by *C*. *albicans* increased the survival of these insects compared to the control group (*P* = 0.0001). After that, we investigated the immune mechanisms involved in the protection against *C*. *albicans* infection, evaluating the number of hemocytes and the gene expression of antifungal peptides. We found that *L*. *paracasei* increased the hemocyte quantity (2.38 x 10^6^ cells/mL) in relation to the control group (1.29 x 10^6^ cells/mL), indicating that this strain is capable of raising the number of circulating hemocytes into the *G*. *mellonella* hemolymph. Further, we found that *L*. *paracasei* 28.4 upregulated genes that encode the antifungal peptides galiomicin and gallerymicin. In relation to the control group, *L*. *paracasei* 28.4 increased gene expression of galiomicin by 6.67-fold and 17.29-fold for gallerymicin. Finally, we verified that the prophylactic provision of probiotic led to a significant reduction of the number of fungal cells in *G*. *mellonella* hemolymph. In conclusion, *L*. *paracasei* 28.4 can modulate the immune system of *G*. *mellonella* and protect against candidiasis.

## Introduction

*Candida albicans* is a human commensal yeast that colonizes the gastrointestinal tract in over half of healthy individuals [1]. This yeast is an opportunistic pathogen that can cause severe and recurrent infections of the mucosa, as well as life-threatening systemic infections [2]. The development of mucosal or systemic candidiasis can occur due to hormonal imbalance, over-use of antibiotics or immunosuppression conditions [3]. Candidiasis is frequently associated with a complex interplay between the fungal virulence factors and the host immune system [4]. The host response to *C*. *albicans* is mediated by a rapid activation of the innate immune system in which macrophages, neutrophils and dendritic cells provide primary protective effect via direct antifungal activities, including phagocytosis and release of antimicrobial peptides [4, 5].

Recently, probiotics bacteria have been studied as a potential method to prevent opportunistic infectious diseases due to their ability to stimulate the immune system [6–8]. According to the World Health Organization, probiotics are live microorganisms that confer health benefits on the host when administered in adequate amounts [9]. In this context, several *Lactobacillus* strains have been investigated as potential probiotic bacteria capable of inhibiting the virulence of pathogens and stimulating the immune system [10–16]. Some studies demonstrated that *Lactobacillus* can interact with *Candida* cells in mixed biofilms and inhibit the growth of *C*. *albicans* [17, 18]. In addition, Oliveira et al. [19] verified that *Lactobacillus rhamnosus* ATCC 7469 was able to decrease significantly the proteinase and hemolysin activities that are considered important virulence factors of *C*. *albicans*. Abedin-Do *et al*. [11] showed that different lactobacilli strains can modulate innate and adaptive immune system responses, preventing the initiation and progression of cancer cells. Moreover, other previous studies showed that certain strains of lactobacilli were capable of modulating the expression of several genes involved in the regulation of the immune system [12–16, 20].

Since these studies suggested that *Lactobacillus* strains can exert immunomodulatory effects, the continuous prophylactic use of probiotics to prevent *Candida* spp. infections may be a potential strategy in preventing recurrent infections. In this context, we identified and investigated the ability of potential probiotic strains to prevent *Candida* infections using the model host *Galleria mellonella*. The immune system of *G*. *mellonella* possesses a number of structural and functional similarities to the innate immune system of mammals [21] and is comprised of cellular and humoral components [22]. Their cellular immune response consists of the synthesis and mobilization of immune cells called hemocytes, which can surround and engulf invading pathogens [23]. The humoral element of these larvae consists of the production of a wide range of antimicrobial peptides (AMP) [24, 25]. In addition, *G*. *mellonella* is a facile infection model that has been used as a screening tool prior to investigating responses in vertebrate models [26]. In previous study, we verified that the prophylactic or therapeutic inoculation of *L*. *acidophilus* ATCC 4356 into *G*. *mellonella* infected by *C*. *albicans* reduced the number of yeast cells in the larval hemolymph and increased the survival of these animals [27].

Using *G*. *mellonella* as a model host, we screened different clinical strains of *Lactobacillus* in order to identify new probiotics strains capable of preventing candidiasis. Since *L*. *paracasei* strain 28.4 exhibited the greatest ability to reduce *Candida* infections, we interrogated a number of insect immune responses to evaluate its probiotic activity. Based on these investigations, we were able to describe the specific responses stimulated by *L*. *paracasei* 28.4 that protected *G*. *mellonella* against *C*. *albicans* infections.

## Materials and methods

### Organisms and strains

In this study we used 9 clinical strains of *Lactobacillus* spp. recovered from the oral cavity and 1 reference strain of *C*. *albicans* from the American Type Culture Collection (ATCC 18804). The oral *Lactobacillus* spp. strains were isolated from the saliva of 41 healthy patients at the Department of Biosciences and Oral Diagnosis, Univ. Estadual Paulista/UNESP (São José dos Campos, SP, Brazil) and included: *Lactobacillus paracasei* (n = 5), *Lactobacillus rhamnosus* (n = 3) and *Lactobacillus fermentum* (n = 1) (Table 1). For identification, the chromosomal DNA of each isolate was extracted using a “PureLink^®^ Genomic DNA kit” (Invitrogen, Carlsbad, CA, USA) according to the manufacturer’s instructions. PCR amplification of the intergenic segment between the 16S and 23S rRNA subunits was carried out as described by Song et al. [28]. All the strains were stored as frozen stocks with 25% glycerol at -80°C until used. The Ethics Committee of the Univ. Estadual Paulista/UNESP approved this study (560.479).

**Table 1:** Clinical strains of *Lactobacillus* used in this study.

Species	Strain designation
*L*. *fermentum*	20.4
*L*. *paracasei*	20.3
*L*. *paracasei*	26.1
*L*. *paracasei*	27.1
*L*. *paracasei*	28.4
*L*. *paracasei*	30.1
*L*. *rhamnosus*	5.2
*L*. *rhamnosus*	19.3
*L*. *rhamnosus*	19.9

### Microbial inoculum preparation

*C*. *albicans* cells were grown in YPD medium (1% yeast extract, 2% bacto-peptone, 2% dextrose) overnight at 30°C with agitation. Cells were collected by centrifugation and washed 3 times with phosphate buffered saline (PBS). Yeast cells were counted using a hemocytometer. The cell number was confirmed by determining CFU/mL on YPD plates. *Lactobacillus* spp. were grown in Lactobacillus MRS Broth (Difco, Detroit, USA) for 24h at 37°C in a bacteriological incubator under microaerophilic conditions. Cells were collected by centrifugation and washed 3 times with PBS and, after this, the number of cells in suspension was determined with a spectrophotometer (Eppendorf Biophotometer Plus, Eppendorf, Hamburg, Germany). For the assay with heat-killed (HK) *Lactobacillus* spp., we incubated bacteria at 80°C for 20 min and subsequently plated the cells on MRS agar to ensure that no viable cells remained.

### *G*. *mellonella* survival

For this study, the methodologies described by Mylonakis *et al*. [29] and Vilela *et al*. [27] were used with some modifications. *G*. *mellonella* (Vanderhorst Wholesale, St. Marys, OH) in their final larval stage were stored in the dark and used within 7 days from shipment. Sixteen randomly chosen *G*. *mellonella* larvae with similar weight and size (250–350 mg) were used per group in all assays. Two control groups were included in the assays that form part of this study: one group was inoculated with PBS, and the other received no injection as a control for general viability.

We initially determined the sub-lethal inoculum concentration of *Lactobacillus* by injecting larvae with serial dilutions of the bacteria. For this purpose, different concentrations of each *Lactobacillus* strain (10^5^ to 10^9^ cells/larvae) were inoculated into *G*. *mellonella* through the last left proleg. The larvae were kept on Petri dishes at 37°C and monitored daily for survival.

To evaluate the effects of probiotics on *C*. *albicans* infections, the larvae were pre-infected with *Lactobacillus* by injecting the bacteria (concentration previously determined) through the last left proleg (volume of 10μL). After 1 h, larvae were infected with 10^6^ cells/larvae of *C*. *albicans* suspended in PBS at the last right proleg (volume of 10μL). Larvae were incubated at 37°C and monitored daily for survival. The experimental groups used in this study are presented in Table 2. Among all the strains tested, *L*. *paracasei* 28.4 reached the highest survival rate and it was selected for the subsequent investigations.

**Table 2:** Experimental groups used to evaluate the effects of *Lactobacillus* strains on *Candida* infections.

Groups	1^st^ Injection	2^nd^ Injection
	(Last left proleg)	(Last right proleg)
PBS	PBS	PBS
*C*. *albicans*	PBS	*C*. *albicans*
*Lactobacillus*	*Lactobacillus*	PBS
*Lactobacillus* + *C*. *albicans*	*Lactobacillus*	*C*. *albicans*
No Injection	-	-

### Quantification of *G*. *mellonella* hemocyte

Larvae were pre-infected with *L*. *paracasei* strain 28.4 by injecting the bacteria at the last left proleg. After 1h, larvae were infected with *C*. *albicans* at the last right proleg. Hemocytes were collected from the hemocoel at 4, 8 and 24h post-injection with *C*. *albicans*. Larvae were bled into tubes containing cold, sterile insect physiologic saline (IPS) (150 mM sodium chloride; 5 mM potassium chloride; 100 mM Tris—hydrochloride, pH 6.9 with 10 mM EDTA, and 30 mM sodium citrate). The hemocytes were identified based on cell morphology and quantified using a hemocytometer. The results were averaged from four replicates.

### Analysis of peptide expression

Larval RNA was extracted using TRIzol (Ambion, Inc., Carlsbad, CA, USA) as recommended by the manufacturer at 4, 8, and 24h post-injection of *L*. *paracasei* strain 28.4. In brief, a 2 mL volume of TRIzol was added to a 15 mL tube containing the homogenized frozen tissue of larvae and incubated at room temperature (RT) for 10 min. Subsequently, 400 μL of chloroform (Sigma-Aldrich, St. Louis, MO, USA) was added and the tubes were centrifuged at 12,000 x *g* for 15 min at 4°C. The supernatant was then transferred to a new tube, and 1 mL of isopropanol (Sigma-Aldrich, St. Louis, MO, USA) was added. After centrifugation, the obtained pellet was washed with 70% ethanol (Sigma-Aldrich, St. Louis, MO, USA), centrifuged again, and suspended in 50 μL of nuclease-free water (Ambion Inc., Carlsbad, CA, USA). The concentration, purity and quality of the RNA were verified using a NanoVue Plus spectrophotometer (GE Healthcare Bio-Sciences, Pittsburgh, USA).

The extracted total RNA (1 μg) was transcribed into complementary DNA (cDNA) using the Verso cDNA Synthesis Kit (Thermo Fisher Scientific Inc, Waltham, MA, USA), according to the protocols recommended by the manufacturer. The primers for the genes that encode β-actin and galiomicin were designed by the authors. The primers for the gene encoding gallerymicin were described and used as indicated by Bergin *et al*. [30] (Table 3). The transcribed cDNAs were amplified for relative quantification of the expression of the genes encoding galiomicin and gallerymicin in relation to the concentration of the reference gene (β-actin).

**Table 3:** PCR primer pairs used to amplify regions of the genes involved in the immune system of *G*. *mellonella* and a reference gene.

Gene Name	Sequence 5´- 3´	bp*	Reference
Galiomicin F^a^	TCCAGTCCGTTTTGTTGTTG	123	This study
Galiomicin R^b^	CAGAGGTGTAATTCGTCGCA	123	This study
Gallerymicin F^a^	GAAGATCGCTTTCATAGTCGC	175	Bergin et al. [30]
Gallerymicin R^b^	TACTCCTGCAGTTAGCAATGC	175	Bergin et al. [30]
β-actin F^a^	ACAGAGCGTGGCTACTCGTT	104	This study
β-actin R^b^	GCCATCTCCTGCTCAAAGTC	104	This study

^a^F indicates a forward primer

^b^R indicates a reverse primer

*Base pair (Fragment size)

The qPCR method was applied to evaluate the amount of the cDNA products in the exponential phase of the amplification reaction. As a detection system, the iTaq^™^ Universal SYBR^®^ Green Supermix (Bio-Rad Laboratories, Inc, Hercules, CA, USA) was used in the following reaction mixture: 5 μL of iTaq Universal SYBR Green (2x), 300 nM of the forward primer, 300 nM of the reverse primer, 2 μL of cDNA solution (diluted 1:10) and 2 μL of nuclease-free water (Ambion Inc., Carlsbad, CA, USA), to obtain a final volume of 10 μL in each well of a 96-well plate (Bio-Rad Laboratories, Inc, Hercules, CA, USA). As a negative control for the reaction, all the reagents were added to the last wells of the plates except for cDNA, and the wells were sealed with optical adhesive (Bio-Rad Laboratories, Inc, Hercules, CA, USA). Subsequently, the plate was placed in a CFX96 Touch^™^ Real-Time PCR Detection System (Bio-Rad Laboratories, Inc, Hercules, CA, USA) device. The following cycling parameters were used: 95°C for 2 min for an initial denaturation followed by 40 cycles of 95°C for 15 s and 60°C for 30 s. After the end of the last cycle, the samples were subjected to dissociation (melting) curve analysis, and the absence of any bimodal curve or abnormal amplification signal was observed and analyzed every 0.1°C. The 2^-ΔΔCT^ method was used to analyze the relative changes in gene expression from the quantitative RT-PCR experiment [31].

### Quantification of *Candida* CFU/larvae in *G*. *mellonella* hemolymph

For this study, the methodology described by Vilela *et al*. [27] was used with some modifications. Larvae were infected with the same method used for the *Galleria mellonella* survival assay. For quantification of the presence of *C*. *albicans* in infected *G*. *mellonella*, the larvae were euthanized 4, 8 and 24 h after infection in the following groups: PBS + *C*. *albicans* and 28.4 + *C*. *albicans*. A pool of 4 larvae was used per group and time. The experiment was carried out in triplicate using 16 larvae per group, for a total of 96 infected larvae. A control group was included for each time point, which was injected with 10 μL PBS into the last left proleg.

At each time point, the larvae were cut in the cephalocaudal direction with a scalpel blade and squeezed to remove the hemolymph, which was transferred to an Eppendorf tube. Serial dilutions were prepared from the hemolymph pool, seeded onto Petri dishes containing Sabouraud dextrose agar (Difco, Detroit, USA) supplemented with chloramphenicol (100 μg/mL), and incubated for 48 h at 37°C. After this period, the colonies were counted for the calculation of CFU/larvae.

### Statistical analysis

Percent survival and killing curves of *G*. *mellonella* were plotted and statistical analysis was performed by the Kaplan-Meier test. Analysis of variance (ANOVA) and Tukey test were used to compare the results obtained in the data of hemocyte count and in the analysis of gene expression. Student’s t-test was used to evaluate the number of *Candida* in the hemolymph of larvae (CPU/larvae). All the tests were performed using GraphPad Prism statistical software (GraphPad Software, Inc., California, CA, USA) and a *P* value ≤ 0.05 was considered significant.

## Results

### Effects of *Lactobacillus* spp. on experimental candidiasis

In order to evaluate the hypothesis that bacteria of the genus *Lactobacillus* have immunomodulatory effects and to identify potential probiotic strains for the prevention of *Candida* infections, we analyzed different *Lactobacillus* clinical strains from our collection including some strains of *L*. *fermentum*, *L*. *paracasei* and *L*. *rhamnosus*.

**Fig 1:**
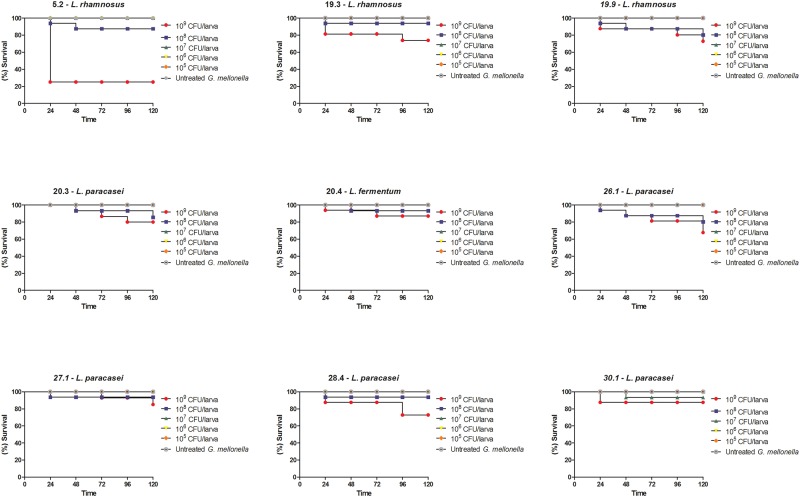
Susceptibility of *G*. *mellonella* to infection with *Lactobacillus* spp. using larvae not infected by *C*. *albicans*. *G*. *mellonella* larvae were treated with serial concentrations of different *Lactobacillus* strains (CFU/larva). The control group was composed of untreated *G*. *mellonella* larvae that received only PBS injection.

Initially, we evaluated the susceptibility of *G*. *mellonella* to *Lactobacillus* strains using larvae not infected by *C*. *albicans* to determine the sub-lethal inoculum concentration. We tested concentrations ranging from 10^5^ to 10^9^ cells/larva and observed larval death only at the two highest concentrations (10^8^ and 10^9^ cells/larvae) (Fig 1).

**Fig 2:**
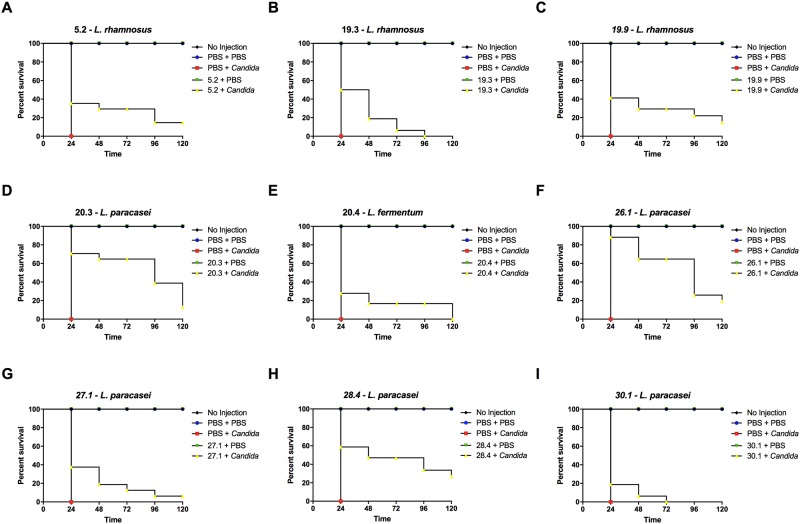
*Lactobacillus* spp. prolongs the survival of *G*. *mellonella* larvae infected with *C*. *albicans*. There was a significant difference between the “*Lactobacillus* strain + *C*. *albicans* group” and “PBS + *C*. *albicans* control group”: **A.** *p* = 0.0097; **B.** p = 0.0013; **C.** *p* = 0.0044; **D.** p = 0.0001; **E.** *p* = 0.0245; **F.** p = 0.0001; **G.** *p* = 0.0075; **H.** p = 0.0003 and **I.** *p* = 0.0733. Kaplan-Meier test, *p*≤ 0.05.

Based on these results, a sub-lethal concentration of 10^6^ cells/larva was adopted for the study to determine the effects of *Lactobacillus* strains on experimental candidiasis. We screened 9 clinical lactobacilli isolates for their ability to prolong longevity of *G*. *mellonella* infected by *C*. *albicans*. In the control group, the infection with *C*. *albicans* without previous injection of lactobacilli caused death in 100% of the larvae within 24h. When the larvae were pretreated with *Lactobacillus* spp. prior to *C*. *albicans* infection, the survival rate of *G*. *mellonela* larvae increased significantly. However, this effect was dependent on the *Lactobacillus* strain injected. More specifically, among the 9 strains analyzed, 6 resulted in the prolonged survival of larvae infected with *C*. *albicans* by up to 120h (Fig 2) and *L*. *paracasei* strain 28.4 reached the greatest survival rate (27%) compared to the other strains (6 to 19%) (Table 4). Based on these findings, this strain was selected for all subsequent assays.

**Table 4:** Effects of *Lactobacillus* spp. on experimental candidiasis based on the analysis of survival curves of *G*. *mellonella* larvae.

Strain	Survival after 120h (%)
20.4 –*L*. *fermentum*	0
20.3 –*L*. *paracasei*	12
26.1 –*L*. *paracasei*	19
27.1 –*L*. *paracasei*	6
28.4 –*L*. *paracasei*	27
30.1 –*L*. *paracasei*	0
5.2 –*L*. *rhamnosus*	14
19.3 –*L*. *rhamnosus*	0
19.9 –*L*. *rhamnosus*	14

**Fig 3:**
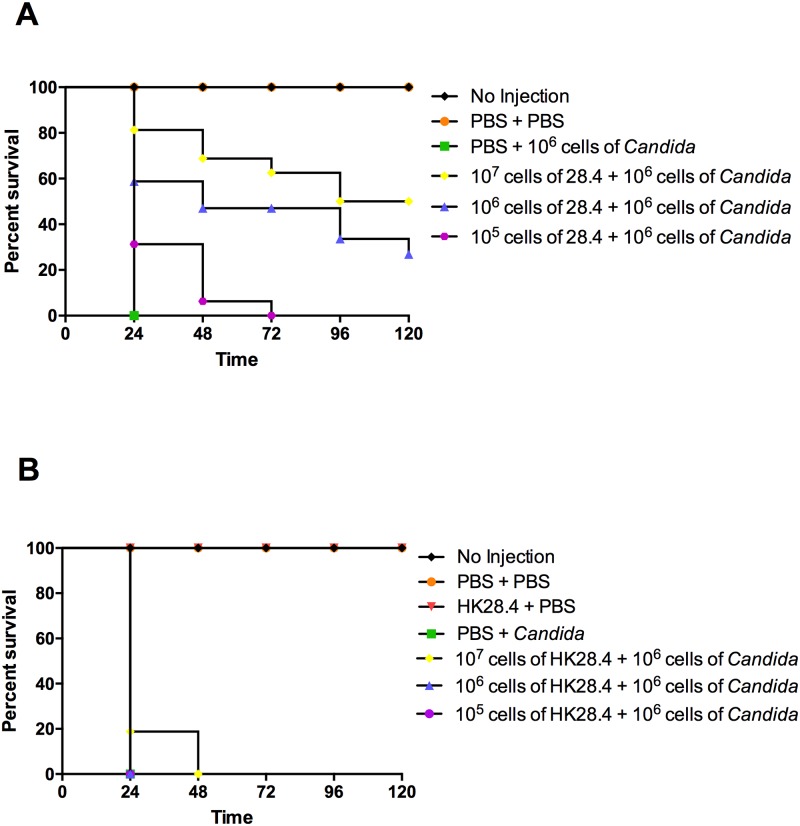
*L*. *paracasei* strain 28.4 prolonged survival of *G*. *mellonella* larvae infected with *C*. *albicans*. **A.** Survival rate for different concentrations of *L*. *paracasei*: significant differences were observed for the groups 10^5^ cells of 28.4 + *Candida* (*p* = 0.0166), 10^6^ cells of 28.4 + *Candida* (*p* = 0.0003) and 10^7^ cells of 28.4 + *Candida* (*p* = 0.0001) in relation to the control group (PBS + 10^6^ cells of *Candida*). **B.** Survival rate for Heat-Killed (HK) *L*. *paracasei*: no significant statistically differences were observed for the groups 10^5^ cells of HK28.4 + *Candida* (*p* = 1.000), 10^6^ cells of HK28.4 + *Candida* (*p* = 1.000) and 10^7^ cells of HK28.4 + *Candida* (*p* = 0.0733) when compared to the control group PBS + *Candida*. Kaplan-Meier test, p≤ 0.05.

In order to determine whether different concentrations of *L*. *paracasei* strain 28.4 could influence the survival rate of larvae infected with *C*. *albicans*, the larvae were pretreated with *L*. *paracasei* at concentrations of 10^5^−10^7^ cells/larva. We observed a dose dependent survival rate of the larvae, whereby an inoculum of 10^7^ cells/larva of *L*. *paracasei* reached higher survival rate in relation to the other concentrations (10^5^ and 10^6^ cells/larvae) (Fig 3A). It was also observed that the increasing *L*. *paracasei* concentration was correlated with a decrease of the melanization of *G*. *mellonella*, that is part of the infection process with *C*. *albicans* (S1 Fig).

In addition, we evaluated if the survival rate of *G*. *mellonella* could be influenced by the viability of *L*. *paracasei* strain 28.4. In this series of experiments, we used the same groups described above, but live *L*. *paracasei* was replaced by heat-killed *L*. *paracasei*. Interestingly, heat-killed *L*. *paracasei* did not provide prophylactic protection, and thus did not increase the survival *G*. *mellonella* infected with *C*. *albicans*. These data indicate that the probiotic action was a consequence of the living and not dead bacteria (Fig 3B).

### Effects of *L*. *paracasei* strain 28.4 on *G*. *mellonella* hemocyte count

**Fig 4:**
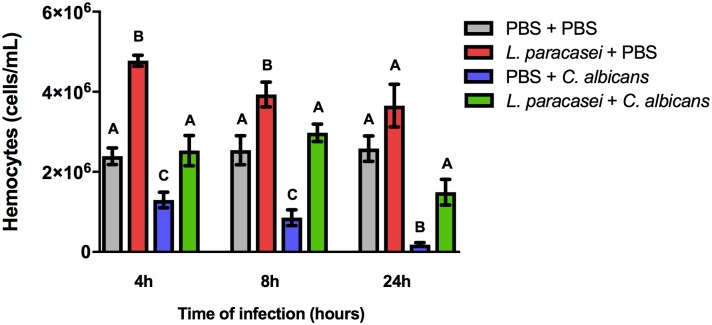
*G*. *mellonella* hemocyte number increased with the injection of *L*. *paracasei* strain 28.4. The group of *L*. *paracasei* 28.4 + PBS increased the hemocyte number compared to a PBS control (PBS + PBS) at all different time points studied. The group *L*. *paracasei* + *C*. *albicans* also increased the hemocyte quantity compared to *C*. *albicans* group (PBS + *C*. *albicans*). PBS + *C*. *albicans* group showed a reduction of hemocyte quantity in relation to the PBS control group, but when the larvae were pretreated with *Lactobacillus* (*L*. *paracasei* + *C*. *albicans* group) the hemocyte quantity was very similar to the values found in the PBS control group. The four groups were compared in each time point studied by ANOVA test (4h: *p* = 0.0001, 8h: *p* = 0.0003, 24h: *p* = 0.0006). The results of Tukey test are indicated by letters: different letters (A, B, and C) represent statistically significant differences among the groups for each time point studied. A p ≤ 0.05 value was considered significant.

To investigate the immune mechanisms associated with the preventive effects of *L*. *paracasei* against *C*. *albicans* infection, we determined the number of available hemocytes in the hemolymph of larvae after 4, 8 and 24h of *Candida* injection. As the higher survival rate of *G*. *mellonella* was achieved with a concentration of 10^7^ cells/larvae of *L*. *paracasei*, we used this concentration to carry out the hemocyte counting assay. Firstly, we analyzed only the larvae not infected by *C*. *albicans* and it was observed an increase in the number of hemocyte in the *L*. *paracasei* group compared to the PBS control group at all different time points studied (4h: 2-fold increase; 8h: 1.54-fold increase and 24h: 1.41-fold increase). In the larvae infected with *C*. *albicans*, the groups pretreated with *L*. *paracasei* also increased the hemocyte number compared to *C*. *albicans* control group in all periods of time (4h: 1.96-fold increase; 8h: 3.49-fold increase and 24h: 8.27-fold increase). Interestingly, we also observed that the PBS + *C*. *albicans* group showed a reduction of hemocyte numbers in relation to the PBS control group, but when the larvae were pretreated with *Lactobacillus* (*L*. *paracasei* + *C*. *albicans* group), the hemocyte quantity was very similar to the values found in the PBS control group (Fig 4). These results indicate that *C*. *albicans* suppresses the hemocyte count and pre-treatment with *L*. *paracasei* strain 28.4 increases the number of circulating hemocytes into the hemolymph, which may protect *G*. *mellonella* from *Candida* infections.

### Effects of *L*. *paracasei* strain 28.4 on the expression of the gene encoding gallerymicin and galiomicin

The presence of an increased hemocyte count suggests that *L*. *paracasei* strain 28.4 may modulate the immune response of *G*. *mellonella* larvae. Thus, we further explored alterations in the immune response examining the expression of antifungal peptides. Using RT-PCR, we evaluated the change in expression of the gene encoding galiomicin, a defensin identified in *G*. *mellonella*, and gallerymicin, a cysteine-rich antifungal peptide.

**Fig 5:**
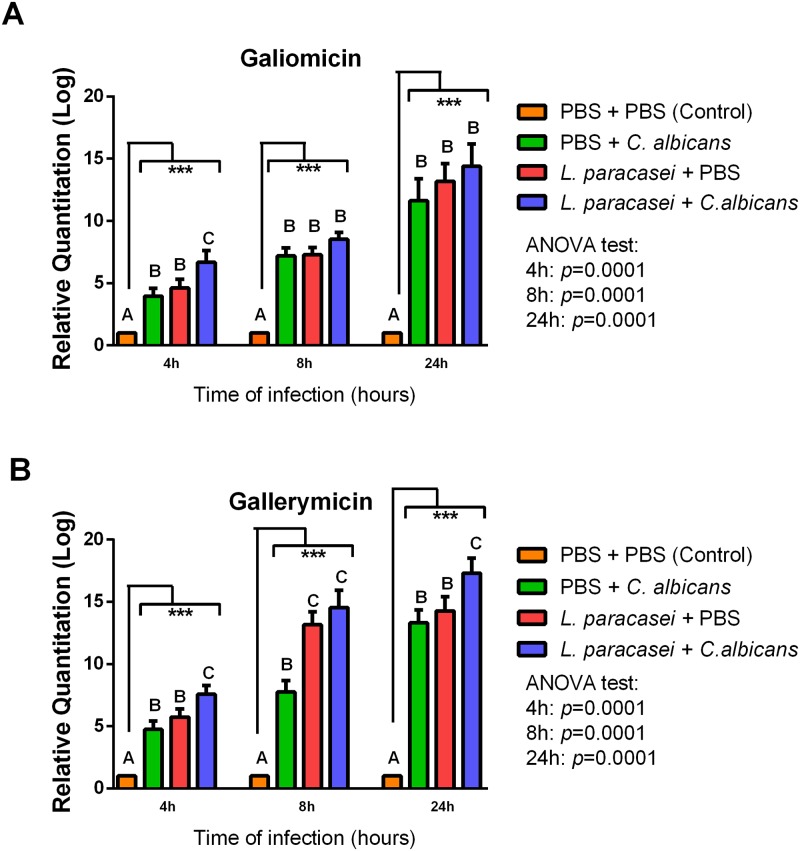
*L*. *paracasei* strain 28.4 increased the expression of antifungal peptides of *G*. *mellonella*. Relative quantification (log) of Galiomicin (**A**) and Gallerymicin (**B**) for the groups treated with only PBS (Control), pre-treated with PBS and infected with *C*. *albicans*, only treated with *L*. *paracasei*, and pre-treated with *L*. *paracasei* and infected with *C*. *albicans*. The units in the Y-axis were calculated based on the 2^-ΔΔCT^ method, and they are expressed as the means and standard deviation. Each gene was normalized and compared with the expression of insects exposed to the control (PBS) using the reference gene β-actin. Different letters (A, B, and C) represent statistically significant differences among the groups. ANOVA and Tukey Tests (*p*≤0.05). ***p ≤ 0.001.

We found that *L*. *paracasei* strain 28.4 was able to increase the expression of both antifungal peptides analyzed. For the gene encoding galiomicin, the group pretreated with *L*. *paracasei* and then infected with *C*. *albicans* had a statistically significant increase (*p* = 0.037) in relation to the control group infected by *C*. *albicans* (PBS + *C*. *albicans*) only for the observation time of 4h. *L*. *paracasei* induced an increase in gene expression of 6.67 and 1.68-fold compared, respectively, to the control group formed by PBS + PBS and the control group composed by PBS + *C*. *albicans* (Fig 5A).

For the gene encoding gallerymicin, the group pretreated with *L*. *paracasei* and then infected with *C*. *albicans* had a greater increase in gene expression compared to the control group infected by *C*. *albicans* (PBS + *C*. *albicans*) for all the times evaluated: 4h (*p* = 0.009), 8h (*p* = 0.0001) and 24h (*p* = 0.0035). *L*. *paracasei* increased the expression of the gene encoding gallerymicin according to observation time, achieving 17.29-fold of increase compared to control group formed by PBS + PBS and 1.87-fold compared to control group formed by PBS + *C*. *albicans* after 24h (Fig 5B). These data show that the pre-treatment with *L*. *paracasei* strain 28.4 increases the level of some antimicrobial peptides, which may act against *C*. *albicans* in the *G*. *mellonella* model.

### Effects of *L*. *paracasei* strain 28.4 on *Candida* CFU in the hemolymph of *G*. *mellonella*

**Fig 6:**
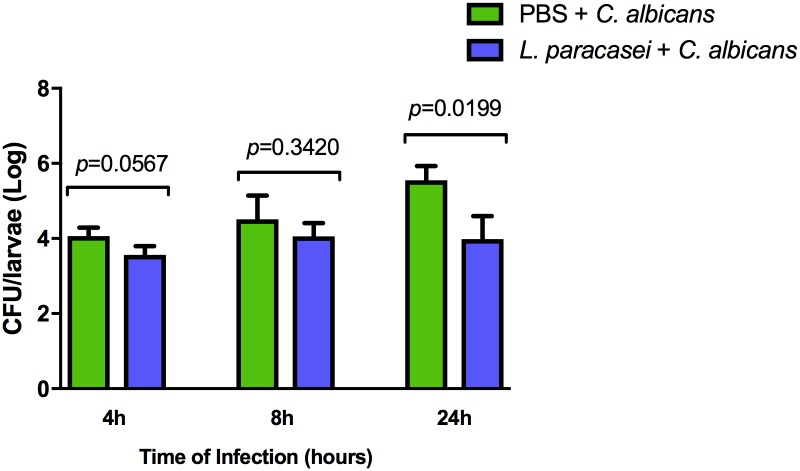
*L*. *paracasei* strain 28.4 decreased the number of fungal cells in *G*. *mellonella* hemolymph. Mean and standard deviation of *C*. *albicans* counts (CFU/larvae) in the hemolymph of *Galleria mellonella* after 4, 8 and 24 h of experimental infection. The following groups were compared at each time of infection: PBS + *C*. *albicans* (control) and *L*. *paracasei* + *C*. *albicans*. A significant difference between groups was only observed after 24 h of infection, with a larger number of CFU/larvae in the control group compared to the *L*. *paracasei* + *C*. *albicans* (*p* = 0.0199). Student *t* test, *p* ≤ 0.05.

The study of *G*. *mellonella* hemolymph culture revealed lower growth of *C*. *albicans* in the groups pretreated with *L*. *paracasei* and infected with *C*. *albicans* compared to *C*. *albicans* control group at all time points studied. A significant difference between groups was only observed at 24 h of infection, with higher growth of *C*. *albicans* in the control group (5.54 Log) compared to the group pretreated with *L*. *paracasei* (3.98 Log) (Fig 6).

## Discussion

There are many studies in the literature describing the probiotic properties of different lactobacilli and their ability to inhibit the colonization of pathogenic microorganisms, to produce biosurfactants and hydrogen peroxide and to modulate the host immune response [10, 32–34]. In this study, we screened new potential probiotic strains of *Lactobacillus* spp. capable of preventing *Candida* infections in a *G*. *mellonella* invertebrate host model. We found that *L*. *paracasei* 28.4 strain improved the survival of *G*. *mellonella* infected with a lethal inoculum of *C*. *albicans*. Our results demonstrated that the immune response of *G*. *mellonella* can be stimulated with a prophylactic provision of probiotic bacteria, making them more resistant to virulent pathogens. These effects were associated with recruitment of hemocytes into the hemolymph and by stimulating antimicrobial peptide response.

Prior to the study of the effects of lactobacilli on the development of candidiasis, we evaluated the susceptibility of *G*. *mellonella* to *Lactobacillus* strains in larvae not infected by *C*. *albicans*. We observed that the strains did not cause death of the animals in concentrations up to 10^7^ cells/larvae, demonstrating low pathogenicity in the *G*. *mellonella* model. There are few studies that used the *G*. *mellonella* model to study probiotic bacteria, in which the strains of bacteria are not also virulent for this host model [27, 35]. It was observed that *Lactobacillus acidophilus* ATCC 4356 [27] and *Lactococcus lactis* NZ9000 [35] were non-pathogenic to *G*. *mellonella* since no larvae died after the injection of bacterial inoculum at concentration of 10^7^ cells/larvae.

By collecting clinical *Lactobacillus* strains from the oral cavity of healthy patients, we found that some natural inhabitant strains provide better protection against *C*. *albicans* than others. We observed that *L*. *paracasei* 28.4 was the best strain to prevent candidiasis in *G*. *mellonella* model. Based on these results, we investigated the capacity of this strain to stimulate the immune system of *G*. *mellonella*. The *G*. *mellonella* model has been successfully used for the study of *C*. *albicans* pathogenesis [36–38]. These invertebrate animals offer a number of advantages over vertebrate models (mice and rats), mainly because they allow the study of strain collections and a large sample number per group without ethical restrictions [36–38]. In contrast to the vertebrate animals, the immune system of insects is not composed of immunoglobulin and immune cells with long-term memory. More specifically, the cellular immune response of *G*. *mellonella* is mediated by hemocytes that represents the main antimicrobial process characterized by phagocytosis [39]. The humoral immune response involves the production of a various antimicrobial peptides (AMP) that can arrest and kill pathogens that evade the cellular immune response [22].

Provision of *L*. *paracasei* 28.4 strain increased the survival rate of *G*. *mellonella* larvae, accompanied by an increase in the number of hemocytes. Taken together, these findings indicate that *L*. *paracasei* is capable to stimulate the cellular immune response of the larvae. Ribeiro et al. [40] also evaluated the anti-*Candida* activity of *L*. *rhamnosus* ATCC 9595 using *G*. *mellonella* as a model host. The treatment with *L*. *rhamnosus* supernatant increased the survival rate of larvae and the hemocytes counting into the hemolymph, suggesting that probiotic strains with antifungal activity can be used as a nondrug method to prevent *Candida* infections.

In our study, we also observed a reduction in quantity of hemocytes in the hemolymph of *G*. *mellonella* larvae after the infection by *C*. *albicans* (PBS + *C*. *albicans* group). Moreover, it was observed that hemocytes levels in the *L*. *paracasei* + *C*. *albicans* group was very similar to the results observed in the PBS + PBS control group. These facts suggest that our prophylactic treatment with *L*. *paracasei* was able to reestablish the hemocyte levels similar to uninfected larvae (PBS + PBS group). Bergin *et al*. [41] performed a study to evaluate whether fluctuations in the number of hemocytes and yeast cells in infected larvae could be used to determine the relative pathogenicity of a range of strains. The results indicated that larvae inoculated with virulent *Candida* strains showed a significant reduction in hemocytes count, while the larvae inoculated with strains with low pathogenicity demonstrated only a slight variation in the number of hemocytes. In their totality, these results confirmed that hemocytes could be used to determine the pathogenicity of microorganisms and modulations of the immune response.

Recently, the laboratory of some of the authors developed a study to evaluate the probiotic action of *L*. *acidophilus* ATCC 4356 in the experimental candidiasis in *G*. *mellonella* [27]. Vilela *et al*. [27] demonstrated that the inoculation of *L*. *acidophilus* into *G*. *mellonella* infected with *C*. *albicans* reduced the number of yeast cells in the larval hemolymph and increased the survival of these animals. These effects can be explained by the results obtained in our current study in which we demonstrated that lactobacilli lead immunomodulation in the *G*. *mellonella* model that may impact in the survival of these animals during fungal infections.

We also explored alterations in the immune response examining the expression of AMP, including the genes encoding gallerymicin and galiomicin. In *G*. *mellonella*, the production of AMP represents the last line of defense. These peptides are released into the hemolymph in order to attack elements of the bacterial or fungal cell wall [23, 42]. AMP are synthesized as pre-proproteins at a rate up to 100 times faster than IgM in mammals and their small size, less than 10 kDa, allows diffusion through the hemolymph to counteract invading pathogens [43]. In general, the mode of action of AMP is through binding to the surface of pathogens that result in damage to the microbial membrane and lead the collapse of the trans membrane electrochemical gradients [44–46].

To the best of our knowledge, this is the first article in the literature that analyzes the AMP expression in *G*. *mellonella* treated with probiotic bacteria. However, quantification of genes expression for gallerymicin and galiomicin is considered an established method that has been used to study the immune system response against *Candida* infections [23, 30, 47]. Bergin *et al*. [30] showed that pre-exposure of *G*. *mellonella* to non-lethal doses of *C*. *albicans* protected against a subsequent lethal infection with *C*. *albicans* due to the increased expression of AMPs. They verified that the maximum expression of AMP occurred between 8 and 24h after administration of the sub-lethal dose of yeast cells. These results agree with our study in which the highest expressions of the genes encoding gallerymicin and galiomicin were found at the times of 8 and 24h.

Once we know the quantity of *C*. *albicans* injected directly into the hemocoel of *G*. *mellonella*, we also evaluated the effects of *L*. *paracasei* on *C*. *albicans* cells present in the hemolymph of these insects at different times of infection (4, 8 and 24h). The results showed that *L*. *paracasei* 28.4 strain affect the number of *C*. *albicans* in the hemolymph at all time points studied. However, a significant difference between the groups was only observed at 24 h of infection. These results corroborate with our data of expression of AMPs, in which we observed higher levels of antifungal peptides expressed in the *G*. *mellonella* in the time of 24 h compared to the other times analyzed. Grounta et al. [20] investigated the effects of *Lactobacillus pentosus* B281, *Lactobacillus plantarum* B282 and *L*. *rhamnosus* GG on *Listeria monocytogenes* and *Staphylococcus aureus* in *G*. *mellonella* model. The authors observed that provision of *Lactobacillus* spp. 6 and 24 h prior to infection by these pathogens affected the survival of infected larvae. Moreover, the number of *L*. *monocytogenes* and *S*. *aureus* in the hemolymph decreased between 1.0 and 1.8 Log compared to the group of infected larvae and non-treated with *Lactobacillus*, respectively. These results indicate that the prophylactic provision of probiotic can be an alternative for the prevention of infectious diseases.

In summary, the clinical strains of *Lactobacillus* isolated from the oral cavity of healthy patients shows varied probiotic activity against *C*. *albicans*. *L*. *paracasei* 28.4 strain represents a new potential probiotic strain that can be used to control *C*. *albicans* infections. In addition, this study indicates that prior exposure to a *L*. *paracasei* dose activates the *G*. *mellonella* immune system, which may allow the larvae to combat a lethal infection by *C*. *albicans*. This effect is mediated by an increase of circulating hemocytes and the production of elevated levels of AMP that consequently reduce *Candida* cells in *G*. *mellonella* hemolymph. This study also demonstrate that *G*. *mellonella* is a suitable model for analyzing specific aspects of broad probiotic immunomodulation.

## Supporting information

S1 Fig Analyses of melanization process after 24h of prophylactic treatment with *L*. *paracasei* and infection with *C*. *albicans*. Control group treated with PBS (1), group treated with 10^5^ cells/larva of *L*. *paracasei* (2), group treated with 10^6^ cells/larva of *L*. *paracasei* (3), and group treated with 10^7^ cells/larva of *L*. *paracasei* (4).
(PDF)
