# Supplementary material for: Lactobacillus paracasei modulates the immune system of Galleria mellonella and protects against Candida albicans infection
Source: PLoS One. 2017 Mar 7;12(3):e0173332. doi: 10.1371/journal.pone.0173332 (PMC5340386; doi:10.1371/journal.pone.0173332)
Supplement: S1 Fig — Control group treated with PBS (1), group treated with 105 cells/larva of L. paracasei (2), group treated with 106 cells/larva of L. paracasei (3), and group treated with 107 cells/larva of L. paracasei (4). (PDF) [file pone.0173332.s001.pdf]

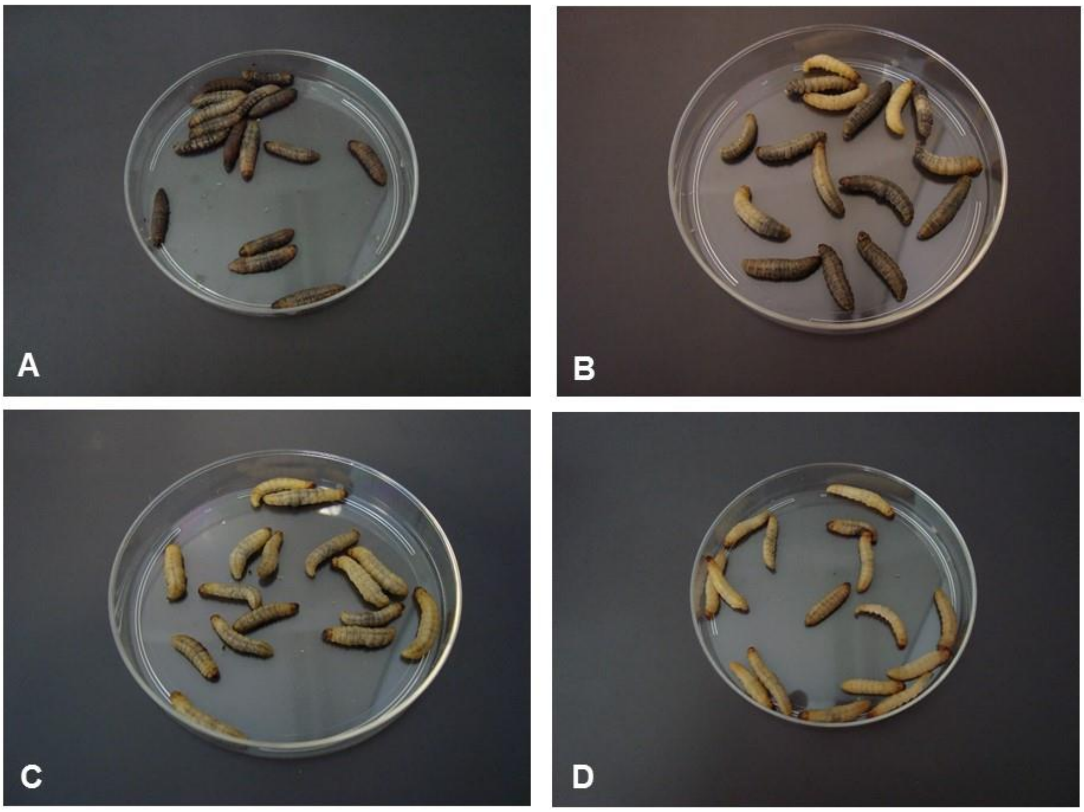

S1 Fig. Analyses of melanization process after 24h of prophylactic treatment with *L. paracasei* and infection with *C. albicans*. Control group treated with PBS (1), group treated with 10^5 cells/larva of *L. paracasei* (2), group treated with 10^6 cells/larva of *L. paracasei* (3), and group treated with 10^7 cells/larva of *L. paracasei* (4).
